# Study on geometrical adaptiveness of pre-bend and swept coupled blades

**DOI:** 10.1016/j.heliyon.2022.e11809

**Published:** 2022-11-23

**Authors:** Quan Wang, Cong Hu, Daode Zhang, Gang Chen, Fengyun Wang

**Affiliations:** a College of Mechanical Engineering, Hubei University of Technology, Wuhan, China; b China Academy of Engineering Physics, Mianyang, China

**Keywords:** Geometrical adaptiveness, Pre-bend/sweep blade, Parameterized model, Alternative BEM theory implementation, Geometrically exact beam theory

## Abstract

Sweep rotor blade would reduce blade fatigue load, but induce additional blade root torsional moment. This paper introduces pre-bend/sweep blade to reduce this additional torsional moment. A parameterized mathematical model is developed to define the geometrical configuration of pre-bend/sweep blade with a fully curvilinear axis based on the curves theory of differential geometry. The blade's geometrical configuration is defined by a series of parameters, thus one can change these parameters to get different blades. An aeroelastic model is established based on the coupling of blade element momentum (BEM) theory and geometrically exact beam theory (GEBT). The BEM theory is implemented in an alternative way to enable it to address the spatial curved and twist blade. In order to investigate the aeroelastic behavior of pre-bend/sweep blade, three kinds of blades are built by the parametrized model and then simulated by the aeroelastic model. From the investigation, it is concluded that pre-bend/sweep blade is better than a purely swept blade for the reason that it shows better performance in reducing the blade root torsional moment as well as alleviating vibration. This paper provides a feasible approach to optimize the geometrical configuration of pre-bend/sweep blade for the purpose of adaptiveness.

## 1 Introduction

The rotor diameter has increased from 30 m for commercial turbines in the early 1990s to above 120 m in recent years. The increasing size of wind turbines can reduce the cost of wind energy. In order to further reduce the cost, the wind turbine industries are talking about next-generation offshore giants of 7.5-12 MW with rotor diameters up to 200 m [1]. The increasing size of wind turbines induces several technical challenges. One of the challenges is the aeroelastic effect of the wind turbine blade, which is caused by the interaction between the blade's aerodynamic loads and structural responses [2]. The aeroelastic effect has to be considered in the design stage of wind turbine blades because of the influence on aerodynamic performance and structural strength. Another challenge is how to alleviate the vibration loads, improve the fatigue performance, and save weight in the rotor design [3]. These challenges need to be overcome when designing the MW-size wind turbine blades. The notion of adaptiveness (also called passive control) [4], [5], [6], proposed decades ago, becomes more potential today on large-scale blades due to their higher flexibility. Therefore, it is a very attractive prospect to the large wind turbine blade's technology in the future. Adaptive blades can control themselves by increasing the coupling between the modes of bend and twist, to alleviate loads and reduce the need for the active control system. The desired effects of coupled deformations can be achieved either by structural or geometrical changes in the blade design. Structural adaptiveness involves modifying the layout and structure of the materials by exploiting the non-conventional couplings that fiber-reinforced laminates tend to possess due to their anisotropic stiffness properties. Lobitz and Griffin had done many researches about structural adaptiveness, see Refs. [7], [8], [9], [10], [11], [12] for detailed descriptions. In this paper, geometrical adaptiveness is studied in detail.

Geometrical adaptiveness is achieved through the shape of blade (i.e. sweeping) to realize coupling of models of deformation (i.e. bending-torsion coupling) [13]. In recent years, many researches about swept blade have been done. Ding Yonggang and Zhang Xing [14] in Tsinghua University optimized swept blades taking both annual energy production and blade root loads into account. Their research showed that annual energy production was increased while torsional moment load was also increased. Pavese C. and Kim T. et al. [15] in DTU (Technical University of Denmark) investigated the relation between different geometric parameters and load alleviations. Their study showed that mildly and purely backward swept shapes were the best option for the design of adaptive blades because they can achieve load alleviations without causing large increases in blade root torsional moment. These researches evidenced that sweep may induce additional blade root torsional moment. This would increase the burden of the pitch actuator. Mehmet Numan Kaya [16] investigated the aerodynamic performance of wind turbines with forward and backward swept blades. It was found that the forward swept blades had the ability to increase the performance while the backward swept blades tend to decrease the thrust coefficient. Therefore, a pre-bend/sweep blade is introduced to counteract the additional torsional moment in our paper.

Unlike the purely swept blade in plan-form, this kind of pre-bend/sweep blade is in spatial form. The three dimensional geometrical configuration for pre-bend/sweep blade is more difficult to be defined. This paper attempts to develop a parameterization model to define the geometrical configuration of pre-bend/sweep blade with a fully curvilinear axis based on the curves theory of differential geometry. With the parameterized model, different blades are obtained by changing the parameters that define the geometric configuration of blades.

As mentioned above, the aeroelastic effect is now being used to help develop adaptive blades by inducing the coupling between modes of bend and twist. As the pre-bend/sweep blade is spatially curved and twisted, the blade's aeroelastic effect is more difficult to be predicted. Both the aerodynamic model and the structural response model have to be able to recognize and deal with this kind of spatial curved and twist blade. An aeroelastic model based on the coupling of BEM theory and GEBT is established. The BEM theory is implemented in an alternative way to enable it to recognize the spatial curved and twist blade. The GEBT, originally proposed by Hodges and his collaborators [17], [18], [19], can recognize spatial curved and twist blade, and can provide sufficient accuracy to model the nonlinear large deformation. Lin Wang et al. [20] used the geometrically exact beam theory to establish an aeroelastic model named NAM_WTB, which was verified to be sufficient accurate for structure dynamic simulation.

**Figure 1:**
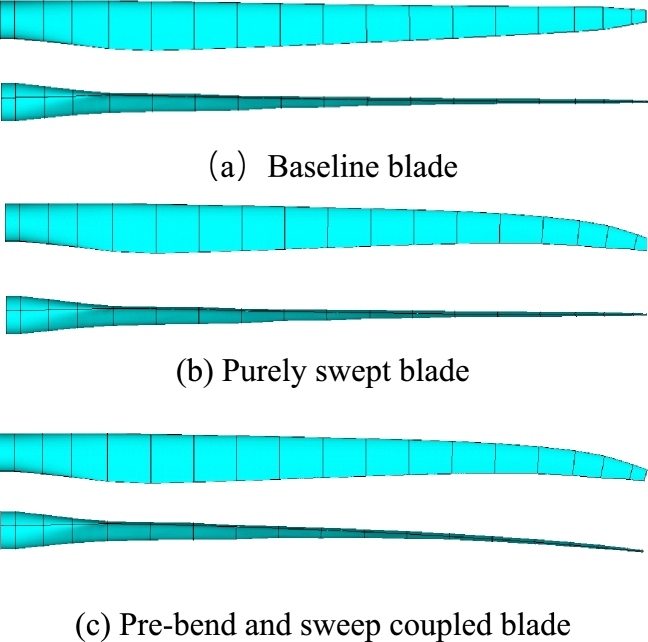
Geometry of the three kinds of blades.

**Figure 2:**
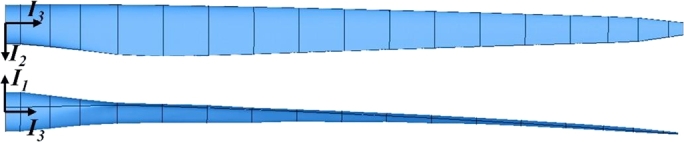
Geometrical configuration of initial curved blade.

**Figure 3:**
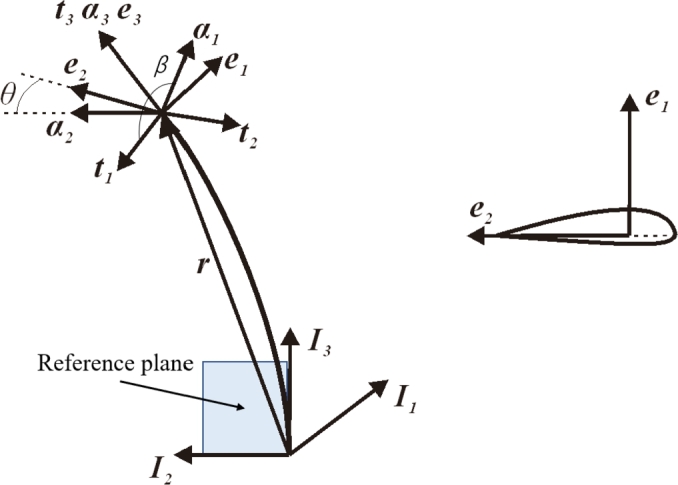
Orthogonal triads of basis vectors used to define the curved reference line.

In order to investigate the advantages of the pre-bend/sweep blade on alleviation of vibration and on reduction of blade root torsional moment, three kinds of blades are built by the parameterized model and then simulated by the aeroelastic model. They are: 1) Baseline straight blade (see Fig. 1 (a)); 2) Purely swept blade (see Fig. 1 (b)); 3) Pre-bend/sweep blade (see Fig. 1 (c)). Compared analysis among their simulation results is made to prove the performance of pre-bend/sweep blade. The geometry of the three blades is shown in Fig. 1.

This paper is arranged as follows. Section 2 describes the parameterized mathematical model; Section 3 mentions the aeroelastic model; Section 4 analyzes the results of the aeroelastic simulation for the three kinds of blades; Section 5 summarizes the important conclusions.

## 2 Parameterization model for pre-bend/sweep blade

Sweep was introduced in fixed-wing aircraft to lower the apparent Mach number and thus the drag at divergence [21], [22]. It was introduced into wind turbine field for purpose of reducing the blade's loads. Pre-bend technology was originally used to increasing wind turbine tower clearance. Many researches about sweep and pre-bend were done separately. However, pre-bend/sweep blade has rarely been studied due to the fact that their three dimensional geometrical configurations are difficult to be defined since they are spatially curved and twisted, despite they may have more capability on geometrical adaptiveness. The current study attempts to develop a parameterized mathematical model of pre-bend/sweep blade based on differential geometric curve.

The geometrical configuration of spatial curved and twist blade can be finally determined if all their sections' three dimensional locations and orientations are defined. Based on this principle, the blade's geometrical configuration is defined within two steps: 1) a curve to be the reference line (blade axis) using Bezier curves; 2) the section's orientation with respect to (w.r.t.) the reference line using rotation matrix. The reference line represents the pre-bend and sweep. The rotation matrix represents the basic aerodynamic parameter (i.e. twist angle). In the second step, it is stipulated that the reference line through the section's shear center perpendicularly.

As we knew, Bezier curves are widely used in computer aided geometric design. They are suitable to express the sections of blade by the reason that the blade root can be described by the parameter 0 and the blade tip can be described by the parameter 1. The parametric equation of Bezier curve is given by formula (1):

(1) $$p(u)=\sum_{k=0}^{n}\frac{n!}{k!(n-k)!}u^{k}{\left(1-u\right)}^{n-k}p_{k}$$

where *n* is the degree of the curve which has n+1 points; $p_{k}$ are the control points which govern the curve; *u* is the abovementioned parameter that runs from 0 to 1. The reference line can be expressed by formula (2):

(2) $$r=xI_{1}+yI_{2}+zI_{3}$$

where $I_{1}$, $I_{2}$ and $I_{3}$ are orthogonal triad of basis vectors. They are located at blade root (see Fig. 2) and the reference coordinate system is defined (see Fig. 3). $I_{3}$ is along with the span-wise of blade. A reference plane is formed by $I_{2}$ and $I_{3}$. If neither conning angle nor pitching angle is taken into account, the reference plane is coincided with the rotor plane. The reference plane plays a key role in this parameterization model. *x*, *y* and *z* are the components along each basis vector, where *x* represents the pre-bend, *y* represents the sweep and *z* represents the spanwise location. They are expressed by equation (3):

(3) $$\left\{ \begin{array}{c} x=p_{x}(u)\\ y=p_{y}(u)\\ z=uL \end{array}\right.$$

where $p_{x}(u)$ and $p_{y}(u)$ are Bezier curves, *L* is the blade length.

As you can see in Fig. 3, $I_{1}$, $I_{2}$ and $I_{3}$ are orthogonal triad of basis vectors; $t_{1}$, $t_{2}$ and $t_{3}$ are Frenet coordinate system; $\alpha_{1}$, $\alpha_{2}$ and $\alpha_{3}$ represent an auxiliary coordinate system which is obtained from a rotation of Frenet coordinate system along $t_{\text{3}}$, and assume that $\alpha_{2}$ is parallel to the reference plane and oriented. *β* is the rotation angle. Therefore $\alpha_{3}$ is coincided with $t_{3}$. The section coordinate system is defined by $e_{1}$, $e_{2}$ and $e_{3}$. *θ* is the twist angle.

The *tangent*, *normal* and *binormal* basis vectors of the reference line are described by the Frenet frame's differential formulas [23, page 29], denoted as *t*, *n* and *b*, respectively. The *tangent* basis vector is given by formula (4):

(4) $$t=\frac{r^{\prime }}{\left|r^{\prime }\right|}$$

where ${\left(\;\right)}^{\prime }$ denotes the derivative w.r.t. *u*, || is the norm operator.

The *normal* basis vector is given by formula (5):

(5) $$n=\frac{r^{\prime \prime }\left|r^{\prime }\right|-(r^{\prime }\cdot r^{\prime \prime })r^{\prime }}{\left|r^{\prime }\times r^{\prime \prime }\right|\left|r^{\prime }\right|}$$

Then the *binormal* basis vector is obtained by formula (6):

(6) b=t×n

Another two important properties of curve are the *curvature* (denoted as *κ*) and *torsion* (denoted as *τ*). They are given by formula (7) and (8):

(7) $$\kappa =\frac{\left|r^{\prime }\times r^{\prime \prime }\right|}{{\left|r^{\prime }\right|}^{3}}$$

(8) $$\tau =\frac{\left(r^{\prime },r^{\prime \prime },r^{\prime \prime \prime }\right)}{{\left|r^{\prime }\times r^{\prime \prime }\right|}^{3}}$$

where ($r^{\prime },r^{\prime \prime },r^{\prime \prime \prime }$) is the mixed product of $r^{\prime }$, $r^{\prime \prime }$ and $r^{\prime \prime \prime }$. From equation (4) to (8), the vectors of ***t***, ***n***, ***b***, *κ* and *τ* are fully determined by $r^{\prime }$, $r^{\prime \prime }$ and $r^{\prime \prime \prime }$. This means that the Bezier functions are able to be replaced if $r^{\prime }$, $r^{\prime \prime }$ and $r^{\prime \prime \prime }$ are offered by another method (i.e. polynomials). The frenet coordinate system (see Fig. 3) is defined based on Frenet frame:

(9) $$\left\{ \begin{array}{c} t_{1}=n\\ t_{2}=b\\ t_{3}=t \end{array}\right.$$

According to a direction cosine matrix $C^{eI}$, the rotation matrix from reference coordinate system to frenet coordinate system is defined by:

(10) $$C_{ij}^{tI}=t_{i}\cdot I_{j}\;(i,j=1,2,3)$$

As the frenet coordinate system is depended by the local properties $r^{\prime }$, $r^{\prime \prime }$ and $r^{\prime \prime \prime }$, it can not be used to define the section's orientation directly. Thus an auxiliary coordinate system ($\alpha_{1}$, $\alpha_{2}$ and $\alpha_{3}$) has to be used (see Fig. 3) to express the blade section's orientation. The rotation matrix from Frenet coordinates to auxiliary coordinates can be defined as:

(11) $$C^{\alpha t}=\left[\begin{array}{ccc} \cos\beta \; & \sin\beta \; & 0\\ -\sin\beta \; & \cos\beta \; & 0\\ 0\; & 0\; & 1 \end{array}\right]$$

The auxiliary coordinate system keeps rotating until that $\alpha_{2}$ is parallel to the reference plane and towards left side as shown in Fig. 3, thus one can get the follow equation:

(12) $$\left\{ \begin{array}{c} \alpha_{2}\cdot I_{1}=0\\ \alpha_{2}\cdot I_{2}\geq 0 \end{array}\right.$$

The rotation matrix $C^{\alpha t}$ is given by Eq. (11) and Eq. (12). Then, the auxiliary coordinate system basis vectors are:

(13) $$\alpha_{i}=C^{\alpha t}t_{i}\;\left(i=1,2,3\right)$$

The definition of auxiliary coordinate system makes it enable to ensure that $\alpha_{2}$ is always parallel to the reference plane no matter what the $r^{\prime }$, $r^{\prime \prime }$ and $r^{\prime \prime \prime }$ are. Therefore, the impact of local property of the reference line can be eliminated. Then, the section's orientation can be defined based on the auxiliary coordinate system. The section coordinate system is defined by $e_{1}$, $e_{2}$ and $e_{3}$, which is obtained from a rotation of auxiliary coordinate system along $\alpha_{3}$. The rotation angle is exactly the twist angle. The rotation matrix can be defined as:

(14) $$C^{e\alpha }=\left[\begin{array}{ccc} \cos\theta \; & \sin\theta \; & 0\\ -\sin\theta \; & \cos\theta \; & 0\\ 0\; & 0\; & 1 \end{array}\right]$$

Thus the section coordinate system basis vectors are:

(15) $$e_{i}=C^{e\alpha }\alpha_{i}\;\left(i=1,2,3\right)$$

The section coordinate system basis vectors are able to represent the airfoil's orientation. As shown in Fig. 3, $e_{2}$ is along with chord-wise, and $e_{\text{1}}$ is perpendicular to the chord. Thus, the airfoil is in the plane expanded by $e_{1}$ and $e_{2}$. The origin of the section coordinate system is located at shear center. It is shown from equation (2) to (15) that the blade's geometrical configuration depends on ***r*** and *θ* when airfoil profiles are given in advance. The rotation matrix from reference coordinate system to section coordinate system is:

(16) $$C^{eI}=C^{e\alpha }C^{\alpha t}C^{tI}$$

It is necessary to introduce a term named curvature and twisting vector [24] (denoted as ***k***) which represents the change of section's orientation per unit arc length. In combination with the previous equations (the equations (4)–(16)), the parameter ***k*** is derived to be the formula (17):

(17) $$k=\left(\tau +\frac{\theta^{\prime }+\beta^{\prime }}{\left|r^{\prime }\right|}\right)e_{3}+\kappa C^{et}t_{2}$$

Significantly, ***k*** is the key parameter in representing the degree of bending and twisting of wind turbine blades. It will be used in structure response model of wind turbine blades.

**Figure 4:**
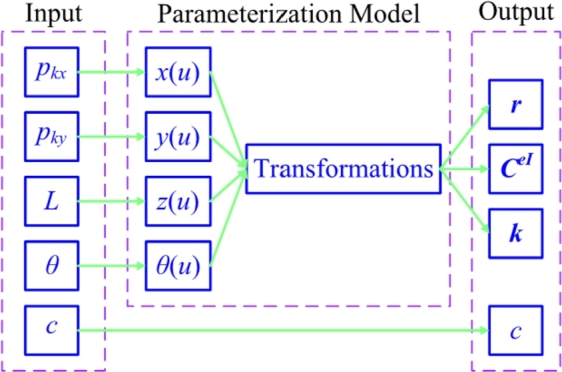
Parameterized model for pre-bend/sweep blade.

According to the equations (1)–(17), now the parameterized process is accomplished. As shown in Fig. 4, the parameterized model of pre-bend/sweep blade is summarized. The input column contains the control points which can express the blade's geometrical configuration. On the other hand, the output column contains the information required by loads computation model and structural response model.

## 3 Aeroelastic model for pre-bend/sweep blade

The aeroelastic model is established based on the coupling of loads computation model and nonlinear structural response model. The loads computation model is composed of aerodynamic model, gravity and centrifugal force model. The nonlinear structural response model is based on geometrically exact beam theory. These two models will be discussed as follows.

### 3.1 Loads computation model

The loads applied on blade include aerodynamic load, gravity and centrifugal force. It is worth to emphasize that both force- and moment-type loads are taken into account.

#### 3.1.1 Aerodynamic model

Even though BEM theory is valid when the wind turbine blade is rigid body, it will be inaccurate considering the deformation of flexible blade. Lago proposed a fine implementation of BEM [25] considering the aeroelastic effect of wind turbine blades. He projected the velocities obtained from momentum theory onto the blade element's plane and then re-projected backwards the resulting forces from blade element theory onto the plane of the stream tube actuator disk. The aerodynamic load calculation of Lago's improved BEM considering aeroelastic effect is carried out in the vector coordinate system. In order to facilitate vector calculation, the induction velocities are used rather than the induction factors.

**Figure 5:**
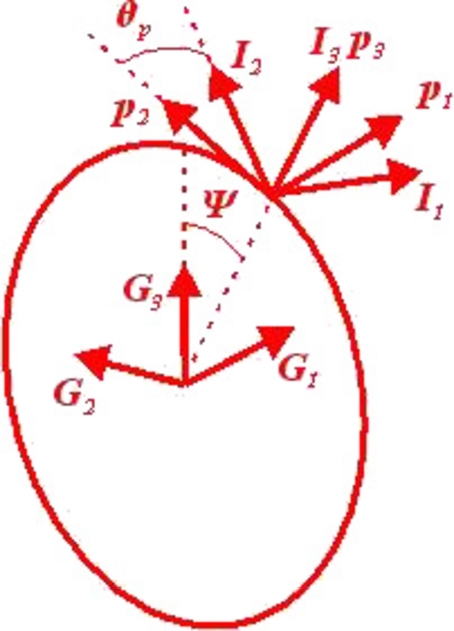
Orthogonal triads of basis vectors attached on rotor hub.

As shown in Fig. 5, the rotor coordinate system is defined by the orthogonal triad of basis vectors. In detail, $p_{1}$ is parallel to the main shaft, and the plane expanded by $p_{2}$ and $p_{3}$ is the rotor plane. The rotation matrix from rotor coordinate system to reference coordinate system is:

(18) $$C^{Ip}=\left[\begin{array}{ccc} \cos\theta_{p}\; & \sin\theta_{p}\; & 0\\ -\sin\theta_{p}\; & \cos\theta_{p}\; & 0\\ 0\; & 0\; & 1 \end{array}\right]\left[\begin{array}{ccc} \cos\theta_{c}\; & 0\; & -\sin\theta_{c}\\ 0\; & 1\; & 0\\ \sin\theta_{c}\; & 0\; & \cos\theta_{c} \end{array}\right]$$

where $\theta_{p}$ is the pitch angle (see Fig. 5). $\theta_{c}$ is the coning angle. The rotor plane as shown in Fig. 5 is coincided with the vertical plane of the rotation axis, therefore $\theta_{c}$ is 0°.

**Figure 6:**
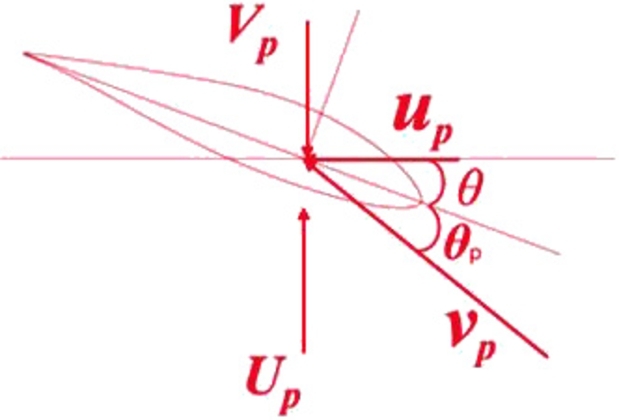
Relative wind speed on the blade section.

The aerodynamic force measured in rotor coordinate system is:

(19) $$f_{p}^{a}=f_{p1}^{a}p_{1}+f_{p2}^{a}p_{2}+f_{p3}^{a}p_{3}$$

where $f_{p1}^{a}$, $f_{p2}^{a}$ and $f_{p3}^{a}$ are the component forces along $p_{1}$, $p_{2}$ and $p_{3}$. Based on momentum theory, the component forces (in introduction velocities formulation) are derived to be:

(20) $$\left\{ \begin{array}{c} f_{p1}^{a}=-4\pi \rho (Fu_{p}\cdot p_{1})\left[(r+r_{hub})\cdot p_{3}\right]\left[(U_{p}+Fu_{p})\cdot p_{1}\right]\\ f_{p2}^{a}=-4\pi \rho (Fu_{p}\cdot p_{2})\left[(r+r_{hub})\cdot p_{3}\right]\left[(U_{p}+Fu_{p})\cdot p_{1}\right]\\ f_{p3}^{a}=0 \end{array}\right.$$

where *F* is the Prandtl tip loss factor; *ρ* is the air density, $r_{hub}$ is the hub radius and ***r*** is obtained in Eq. (2). As shown in Fig. 6, $U_{p}$ is the upcoming wind speed, $u_{p}$ is the induction velocity, $\nu_{p}$ is the relative wind speed in rotor coordinate system, $V_{p}$ is the frame speed due to rotor rotation.

The aerodynamic force measured in section coordinate system is:

(21) $$f_{e}^{a}=f_{e1}^{a}e_{1}+f_{e2}^{a}e_{2}+f_{e3}^{a}e_{3}$$

where $f_{e1}^{a}$, $f_{e2}^{a}$ and $f_{e3}^{a}$ are the component forces along $e_{1}$, $e_{2}$ and $e_{3}$. Based on blade element theory, the component forces are:

(22) $$\left\{ \begin{array}{c} f_{e1}^{a}=0.5\rho c\overset{¯}{v}F_{1}(C_{L}v_{e}\cdot e_{2}+C_{D}v_{e}\cdot e_{1})\\ f_{e2}^{a}=0.5\rho c\overset{¯}{v}F_{1}(C_{D}v_{e}\cdot e_{2}-C_{L}v_{e}\cdot e_{1})\\ f_{e3}^{a}=0 \end{array}\right.$$

where *c* is the chord and $F_{1}$ is the Shen factor [26] which is used here to correct the aerodynamic coefficients near tip regions, where $\overset{¯}{v}$ is the relative speed in section coordinate system. Thus the aerodynamic coefficients $C_{L}$, $C_{D}$ and $C_{M}$ corrected by rotational-augmentation or dynamic-stall effects can be obtained.

Then, the aerodynamic forces obtained by blade element theory are projected onto the rotor plane and made equal to the aerodynamic forces calculated by momentum theory. It can be expressed by formula (23).

(23) $$f_{p}^{a}=\sum_{i=1}^{B}{\left(C^{pe}f_{e}^{a}\right)}_{i}$$

Where *B* is the number of blades; $C^{pe}$ is the rotation matrix from section coordinate system to wind turbine coordinated system.

The aerodynamic moment w.r.t. shear center is given by formula (24):

(24) $$m_{e}^{a}=m_{e3}^{a}e_{3}+\varsigma^{a}\times f_{e}^{a}$$

where $\varsigma^{a}$ is the offset of aerodynamic center w.r.t. shear center measured in section coordinate system; $m_{e3}$ is the moment caused by $C_{M}$ and it is given by formula (25):

(25) $$m_{e3}^{a}=0.5\rho c^{2}{\overset{¯}{v}}^{2}C_{M}$$

The distributed force (per unit length) applied on blade can be obtained by superposition of aerodynamic force, gravity and centrifugal force. Similarly, the distributed moment (per unit length) applied on blade can be obtained by superposition of aerodynamic moment, gravity moment and centrifugal moment in equation (26):

(26) $$\left\{ \begin{array}{c} f_{e}=f_{e}^{a}+f_{e}^{g}+f_{e}^{c}\\ m_{e}=m_{e}^{a}+m_{e}^{g}+m_{e}^{c} \end{array}\right.$$

$f_{e}^{g}$ is the gravity force measured in section coordinate; $f_{e}^{c}$ is the centrifugal force measured in section coordinate; $m_{e}^{g}$ is the gravity moments w.r.t. shear center; $m_{e}^{c}$ is the centrifugal moments w.r.t. shear center.

#### 3.1.2 Verification of aerodynamic model

**Figure 7:**
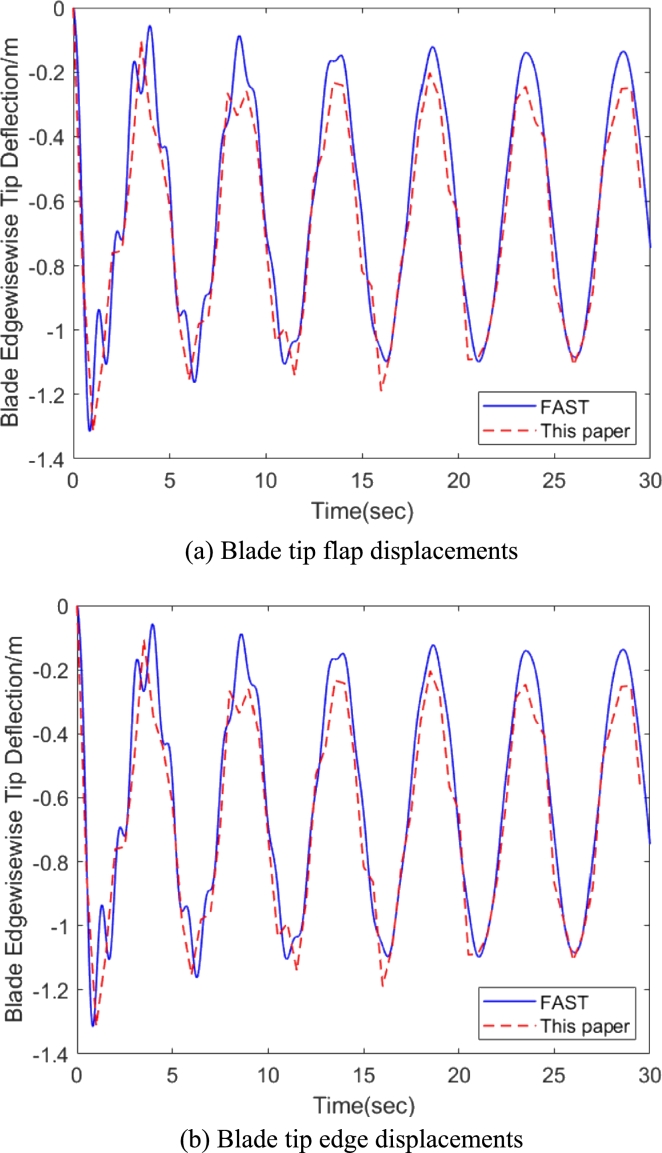
Comparisons of blade displacement between this paper and FAST.

In order to prove the correctness of the method in this paper, the compared analysis was made using NREL 5-MW reference blade in rated wind condition. Details of NREL 5MW wind turbine are in Ref. [27]. The operational parameters are summarized in Table 1. It can be seen from Fig. 7 that the proposed method is in good agreement with the FAST simulated results.

**Table 1:** Basic parameters of NREL 5-MW wind turbine.

Description	Value
Rotor orientation	Upwind
Number of blades	3
Blade length/Hub radius	61.5 m/1.5 m
Rotor pre-cone	2.5 deg
Shaft tilt	5 deg
Rated wind speed	11.4 m/s
Rated rotor speed	12.1 rpm

**Figure 8:**
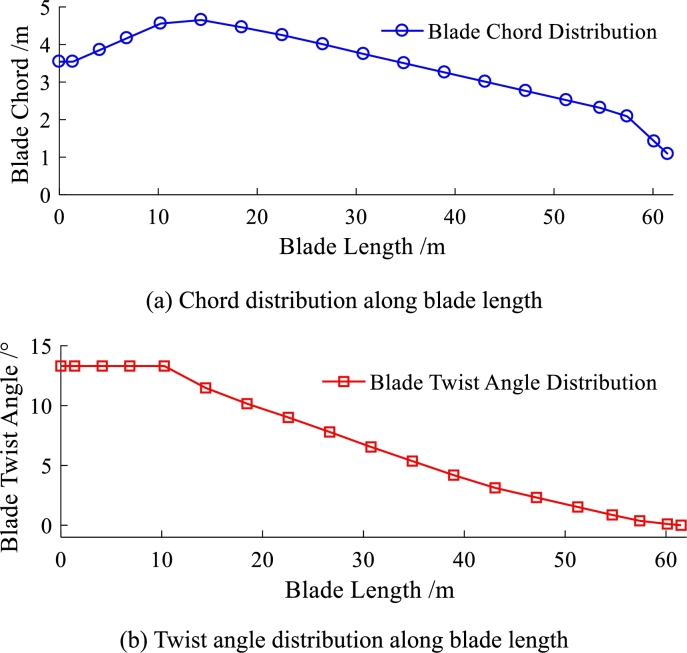
The chord and twist angle distribution of NREL 5MW baseline blade.

**Figure 9:**
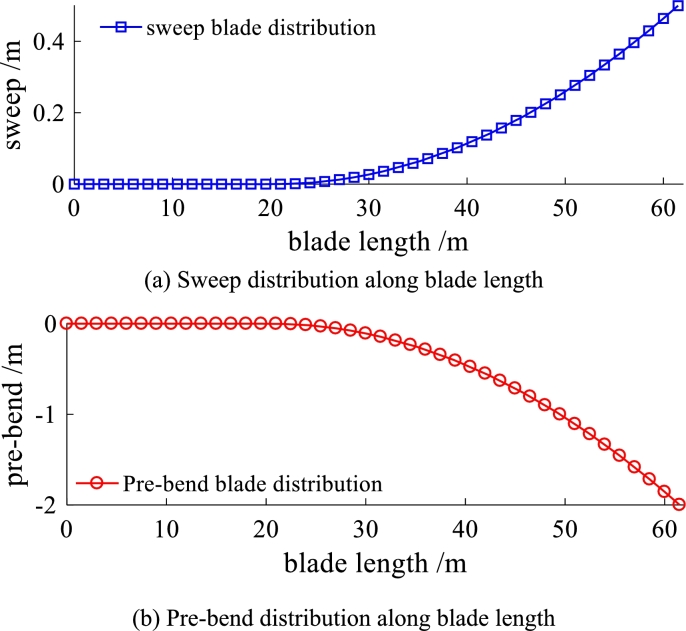
Sweep and pre-bend distribution of the modified blade.

A novel pre-bend/sweep blade is built by using the parameterized model discussed above. The NREL 5MW is chosen to be a referenced wind turbine [28]. Its twist angle (see Fig. 8 (a)) and chord length (see Fig. 8 (b)) remain unchanged. However, the reference line (originally straight) is modified to curve (see Fig. 9, including pre-bend and sweep). According to reference [15], the sweep at the tip is about 0.5% chord length. Usually, the pre-bending is between 2% and 4% chord length. The typical zero yaw-error wind direction for the geometrical configuration of the modified blade is shown in Fig. 2.

**Figure 10:**
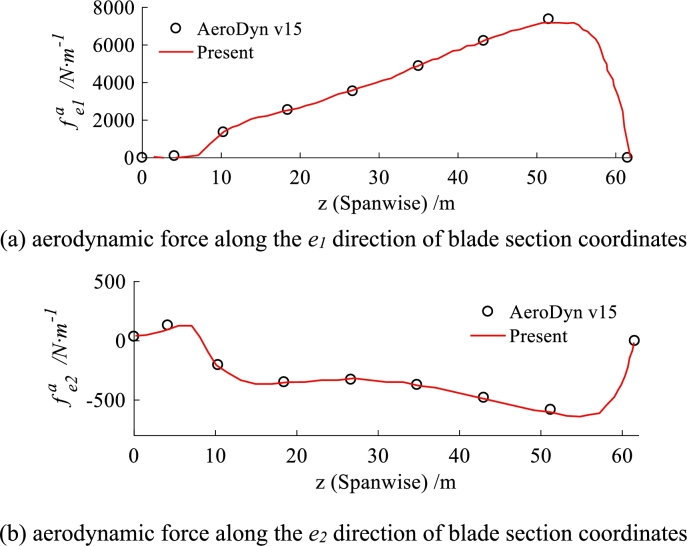
Aerodynamic force calculated by this paper against AeroDyn v15.

The verification is carried out against an existing widely used aerodynamic code named AeroDyn v15 [29], which can also solve the initial curved blade. The verification results are shown in Fig. 10(a) and Fig. 10(b) that the computed aerodynamic forces $f_{e1}^{a}$ and $f_{e2}^{a}$ along $e_{\text{1}}$ and $e_{\text{2}}$ as presented in Eq. (22) are well agreed with AeroDyn v15.

**Figure 11:**
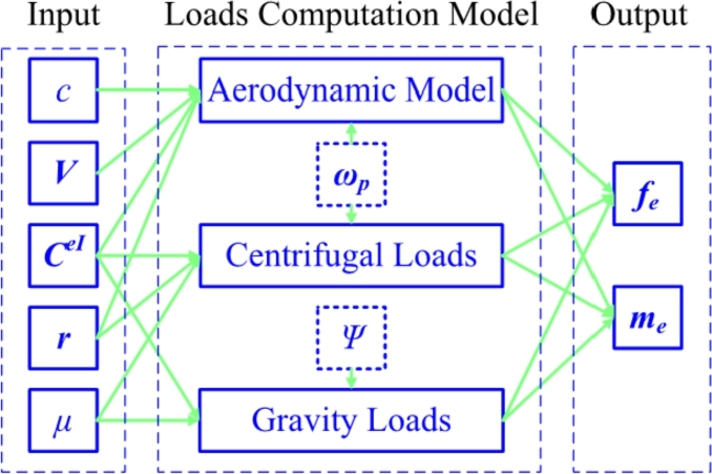
Loads computation model for pre-bend/sweep blade.

According to Equations (18) to (26), the loads computation model is summarized in Fig. 11. The input column contains chord *c*, the blade additional velocity ***V*** produced by vibration, rotated matrix $C^{eI}$, reference line location ***r*** and the blade mass per unit length *μ*. The output column contains the applied distributed loads $f_{e}$ and $m_{e}$.

### 3.2 Nonlinear structural response model of blade

#### 3.2.1 Application of geometrically exact beam theory

According to Kirchhoff thin shell theory and Hodges' relation of blade velocity and displacement, the dynamic control equation of pre-bent/swept blade is derived as follows:

(27) $$\left\{ \begin{array}{c} \frac{\overset{¯}{\partial }}{\partial t}\left\{\begin{array}{c} \gamma \\ \kappa \end{array}\right\}-\frac{\overset{¯}{\partial }}{\partial u}\left(h\left\{\begin{array}{c} V\\ \Omega \end{array}\right\}\right)=\left[\begin{array}{cc} \overset{˜}{k}+\overset{˜}{\kappa }\; & \overset{˜}{e}+\overset{˜}{\gamma }\\ \overset{˜}{0}\; & \overset{˜}{k}+\overset{˜}{\kappa } \end{array}\right]\left\{\begin{array}{c} V\\ \Omega \end{array}\right\}\\ \frac{\overset{¯}{\partial }}{\partial t}\left(M\left\{\begin{array}{c} V\\ \Omega \end{array}\right\}\right)-\frac{\overset{¯}{\partial }}{\partial u}\left(hE\left\{\begin{array}{c} \gamma \\ \kappa \end{array}\right\}\right)=\left[\begin{array}{cc} \overset{˜}{k}+\overset{˜}{\kappa }\; & \overset{˜}{0}\\ \overset{˜}{e}+\overset{˜}{\gamma }\; & \overset{˜}{k}+\overset{˜}{\kappa } \end{array}\right]E\left\{\begin{array}{c} \gamma \\ \kappa \end{array}\right\}\\ \;-\left[\begin{array}{cc} \overset{˜}{\Omega }\; & \overset{˜}{0}\\ \overset{˜}{V}\; & \overset{˜}{\Omega } \end{array}\right]M\left\{\begin{array}{c} V\\ \Omega \end{array}\right\}+\left\{\begin{array}{c} f_{e}\\ m_{e} \end{array}\right\}+\left\{\begin{array}{c} f_{d}\\ m_{d} \end{array}\right\} \end{array}\right.$$

where ***γ*** is the linear strain of blade in section coordinate system; ***κ*** is the change of *curvature and twisting vector* in deformation. ***V*** is the linear velocity in section coordinate system; **Ω** is the angular velocity in section coordinate system; *M* is the mass matrix; ***K*** is the stiffness matrix; ($\overset{˜}{\;}$) is the cross product operator, for example: $\overset{˜}{\kappa }$ is the cross product operator of the change in bending and torsion; $e={\left[\begin{array}{ccc} 0\; & 0\; & 1 \end{array}\right]}^{T}$;

*h* is obtained from replacement of $\frac{\overset{¯}{\partial }(\;)}{\partial s}$ to $\frac{\overset{¯}{\partial }(\;)}{\partial u}$, and it is expressed by formula (28):

(28) $$h=\frac{1}{{\left|r^{\prime }\right|}_{initial}(1+\gamma_{3})}$$

where $\gamma_{3}$ is the extensional strain along $e_{3}$. $f_{d}$ and $m_{d}$ in Eq. (27) are damping force and damping moment calculated by formula (29):

(29) $$\left\{\begin{array}{c} f_{d}\\ m_{d} \end{array}\right\}=-\upsilon \frac{\overset{¯}{\partial }}{\partial t}\left(E\left\{\begin{array}{c} \gamma \\ \kappa \end{array}\right\}\right)$$

where *υ* is the damping coefficient.

For a wind turbine blade, the initial conditions of the dynamic governing equation (Eq. (27)) are expressed by equation (30):

(30) $$\left\{ \begin{array}{c} V(u,t=0)=0\\ \Omega (u,t=0)=0\\ \gamma (u,t=0)=0\\ \kappa (u,t=0)=0 \end{array}\right.$$

The boundary conditions can be set by equation (31):

(31) $$\left\{ \begin{array}{c} V(u=0,t)=0\\ \Omega (u=0,t)=0\\ \gamma (u=1,t)=0\\ \kappa (u=1,t)=0 \end{array}\right.$$

Solving Eq. (27), then one can obtain ***V***, **Ω**, ***γ*** and ***κ*** but without ***r*** or $C^{eI}$.

By integrating the change of *curvature and twisting vector*, then one can get the rotation matrix using the matrix (32):

(32) $$C^{Ie}=\left[\begin{array}{ccc} 2(q_{0}^{2}+q_{1}^{2})-1\; & 2(q_{1}q_{2}-q_{0}q_{3})\; & 2(q_{1}q_{3}+q_{0}q_{2})\\ 2(q_{1}q_{2}+q_{0}q_{3})\; & 2(q_{0}^{2}+q_{2}^{2})-1\; & 2(q_{2}q_{3}-q_{0}q_{1})\\ 2(q_{1}q_{3}-q_{0}q_{2})\; & 2(q_{2}q_{3}+q_{0}q_{1})\; & 2(q_{0}^{2}+q_{3}^{2})-1 \end{array}\right]$$

where ***q*** is a column matrix which contains the Euler parameters, $q\;={\left[\begin{array}{cccc} q_{0}\; & q_{1}\; & q_{2}\; & q_{3} \end{array}\right]}^{T}$.

Then one can obtain the ***r*** with the formula (33):

(33) $$r=\int_{0}^{u}\frac{1}{h}C^{Ie}edu$$

Eq. (27) is the dynamic model for the nonlinear structural response. If static model is needed, one can remove the terms of time and velocity, and then Eq. (27) can be expressed by equation (34):

(34) $$\frac{\overset{˜}{\partial }}{\partial u}\left(hE\left\{\begin{array}{c} \gamma \\ \kappa \end{array}\right\}\right)=-\left[\begin{array}{cc} \overset{˜}{k}+\overset{˜}{\kappa }\; & \overset{˜}{0}\\ {\overset{˜}{e}}_{3}+\overset{˜}{\gamma }\; & \overset{˜}{k}+\overset{˜}{\kappa } \end{array}\right]E\left\{\begin{array}{c} \gamma \\ \kappa \end{array}\right\}-\left\{\begin{array}{c} f_{e}\\ m_{e} \end{array}\right\}$$

Its boundary conditions are reduced to equation (35):

(35) $$\left\{ \begin{array}{c} \gamma (u=1)=0\\ \kappa (u=1)=0 \end{array}\right.$$

**Figure 12:**
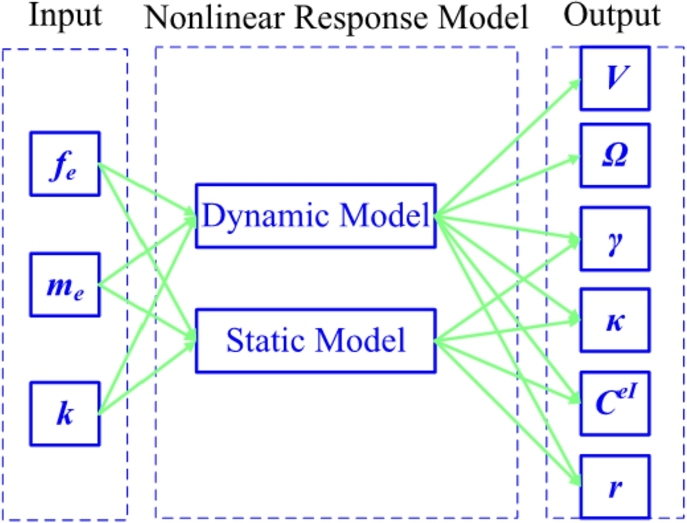
Configuration of nonlinear structural response model.

According to Equations (27) to (35), the nonlinear structural response model is summarized in Fig. 12, where both dynamic and static models are listed. The input column contains the applied loads $f_{e}$, $m_{e}$, and the *initial curvature and twisting vector* ***k***. On the other hand, the output column contains the blade response properties ***V***, **Ω**, ***γ***, ***κ***, $C^{eI}$ and ***r***.

#### 3.2.2 Verification of nonlinear structural response model

This verification is carried out against finite element method software (i.e. ANSYS). It may look like more reasonable that the verification of structural response model is implemented on a full-scale composite laminate wind turbine blade. However, it is not so due to the fact that section properties (i.e. *EI*, *GJ* et al.) of composite laminate beam, especially for complex hollow shell beam, can not be calculated accurately. As GBET is very sensitive to section properties, inaccurate section properties may lead to huge error. For this reason, the verification is feasible to be implemented on a beam, whose section properties can be calculated accurately. Thus a solid isotropic cantilever beam is used as the study case. It has a uniform rectangle cross-section of 0.1×0.1 m, and its value of Young's modulus and Poisson ratio are 2.0E11 Pa and 0.3. The beam is built with the characteristic of spatially curved and twisted by the parameterized method discussed in section 2. Its magnitude of bending is represented by *x* and *y* (see Eq. (3)), and its magnitude of torsion is represented by *θ*. The length of beam along z direction is 4 m. The torsion θ=0.4rad.

**Figure 13:**
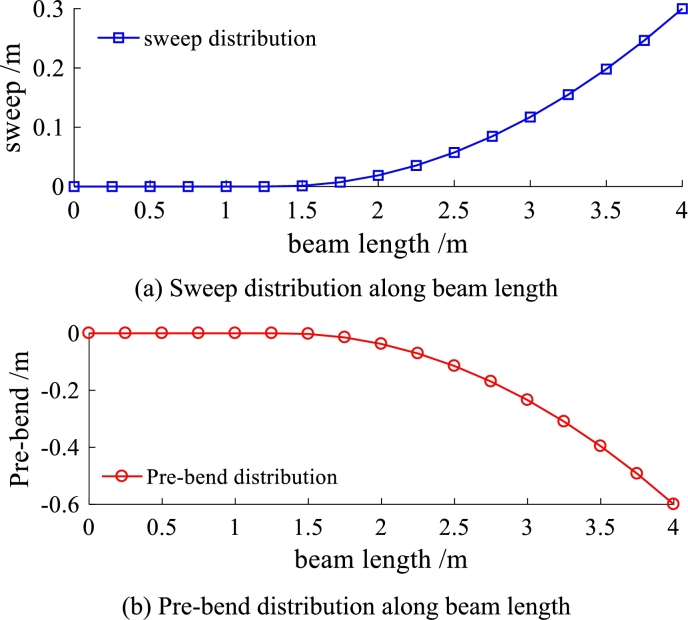
Sweep and pre-bend distribution of the solid isotropic cantilever beam.

**Figure 14:**
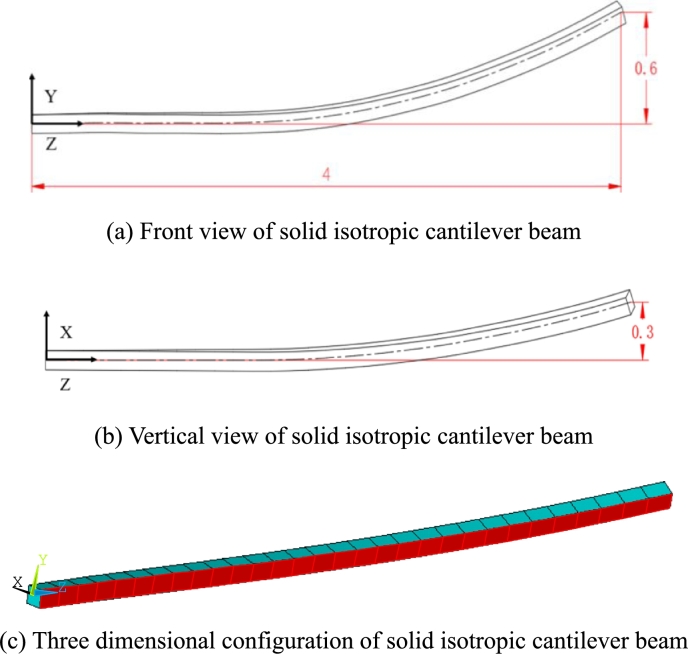
Solid isotropic cantilever beam.

**Figure 15:**
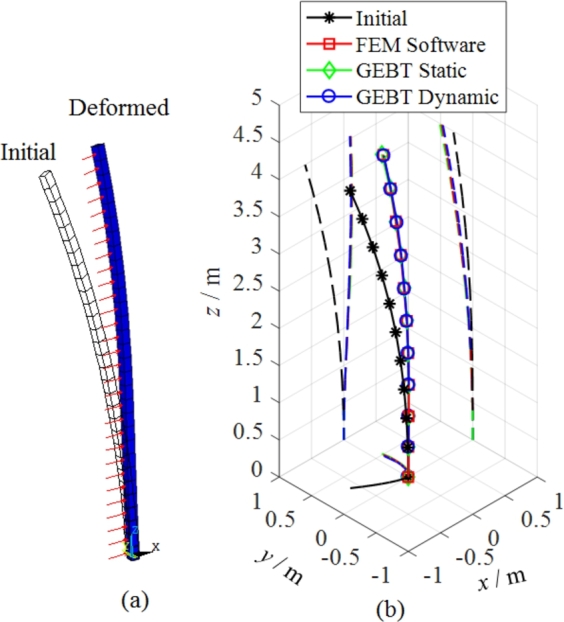
Structural deformations among different approaches.

**Figure 16:**
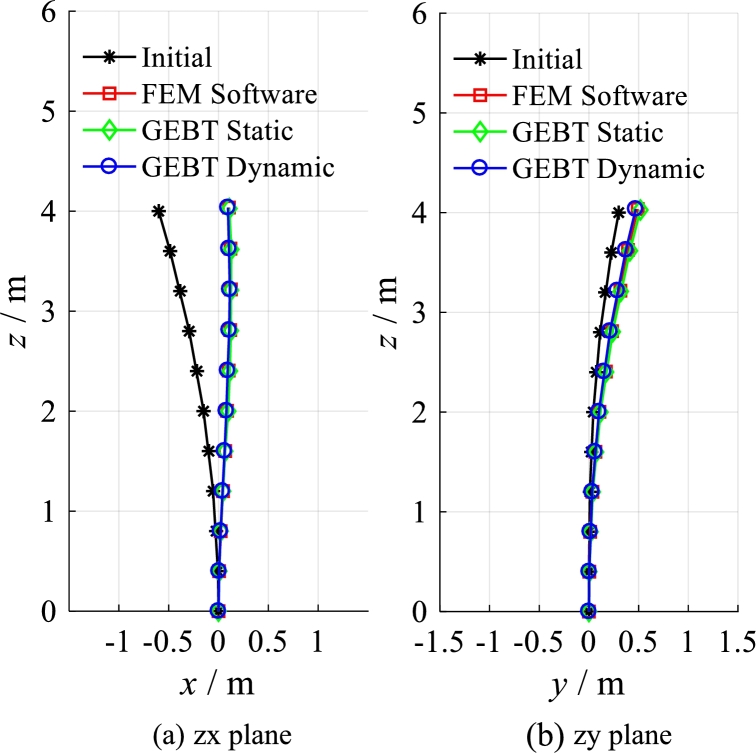
The projection of the beam deformation configuration on the zx and zy plane.

Sweep and pre-bend distribution of the solid isotropic cantilever beam are shown in Fig. 13. The seep distribution along beam length is shown in Fig. 13 (a) and the pre-bend distribution along beam length is shown in Fig. 13 (b). The three dimensional geometrical configuration of the beam is obtained (see Fig. 14(c)). The front view of solid isotropic cantilever beam is shown in Fig. 14 (a) and the vertical view of solid isotropic cantilever beam is shown in Fig. 14 (b). Firstly, the ANSYS software is used to calculate the deformation of the beam, under the uniform pressure (the value is 4.0E5 Pa). The red surfaces in Fig. 14 (c) are the regions where pressure is loaded. The deformed beam is shown in Fig. 15 (a). Then, the beam with the same load case is calculated by the structural response model. Fig. 15 (b) shows the deformed configurations of the beam and their projections (the dashed line). Fig. 16 shows in detail the projection of the beam deformation configuration on the ZX plane (see Fig. 16 (a)) and ZY plane (see Fig. 16 (b)). As demonstrated in Fig. 16, the deformed results of the structural response models (static and dynamic) are able to follow the results of FEM software very well. Therefore, this model is validated to have the ability to predict the structural response for the spatial curved and twist blades.

## 4 Simulations and results

### 4.1 Simulation model

**Figure 17:**
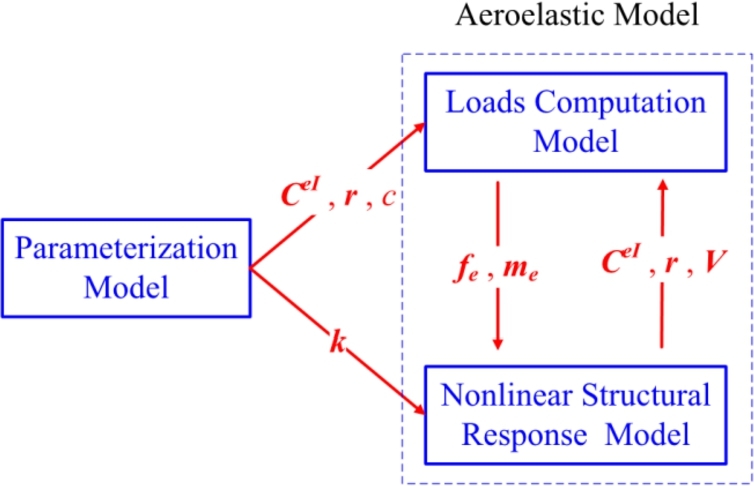
The core flowchart of the simulation model.

The simulation model is based on parametric model and aeroelastic mode. The core part of the simulation model is shown in Fig. 17. Firstly, the pre-bend/sweep blade is built by the parameterization model. Secondly, the geometrical parameters of the pre-bend/sweep blade are passed to the aeroelastic model (both loads computation model and nonlinear structural response model). Thirdly, the distributed loads are calculated in the loads computation model and then passed to the nonlinear structural response model. Lastly, the structural responses behaviors are obtained in the nonlinear structural response model and returned back to the loads computation model. The loop, between the loads computation model and the structural response model, is executed until simulation is converged.

### 4.2 Simulations on NREL 5MW blade

The simulation is made to investigate the vibration and blade root torsional moment for the pre-bend/sweep blade. Three kinds of blades are built based on NREL 5MW wind turbine blades. They are defined as follows:

a) Baseline blade (NREL 5MW blade);

b) Purely swept blade;

c) Pre-bend and swept coupled blade.

**Figure 18:**
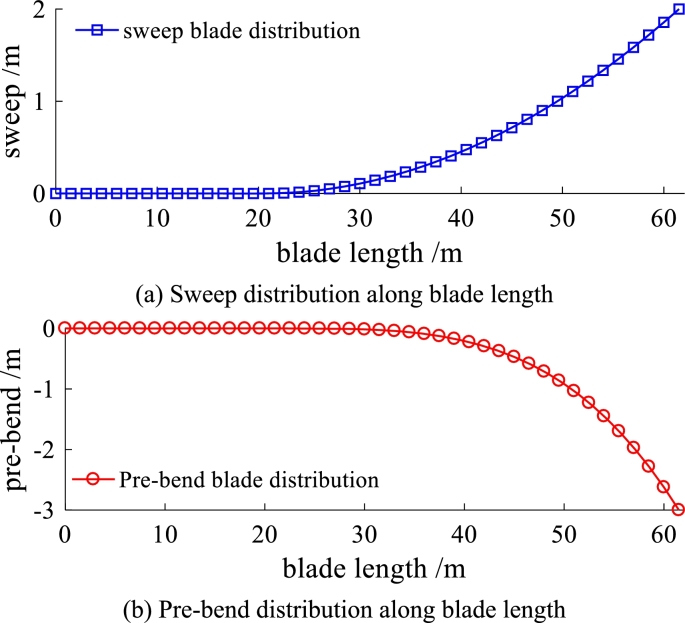
Sweep and pre-bend distribution of modified NREL 5MW blade.

Their chord lengths and twist angles are still the same with NREL 5MW blade (see Fig. 8). The reference line of the baseline blade keeps straight. Whereas, the reference line of purely swept blade is purely swept (in planform). Differently, the reference line of pre-bend/sweep blade is pre-bend and sweep coupled (in spatial-form). It should be noted that the three kinds of blades have the same section property along blade span-wise direction. The magnitudes of pre-bend and sweep are shown in Fig. 18. The sweep distribution along blade length is shown in Fig. 18 (a) and the pre-bend distribution along blade length is shown in Fig. 18 (b). As shown in Fig. 1, the geometry of the third kind of blade exhibits is characterized by pre-bend and sweep.

**Figure 19:**
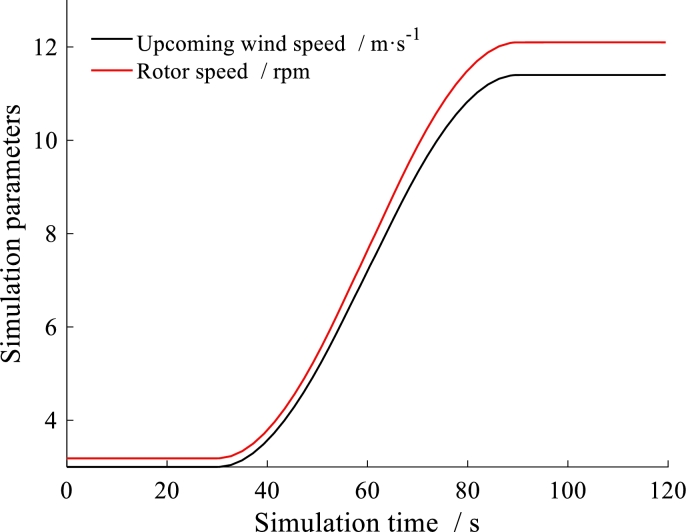
Time evolution of upcoming wind speed and rotor speed.

Fig. 19 shows the time evolution of operational condition during simulation period. The upcoming wind speed accelerates from cut in speed to rated speed with a sinusoidal ramp. The rotor speed follows the wind speed by means of that the tip speed ratio remains at nominal value, until reaches 12.1 rpm when wind speed accelerates to rated speed. The purpose of this setting is to analyze the multidirectional coupled aeroelastic behavior of the wind turbine blade at different wind speeds. The pitch angle remains at zero so that it is not plotted.

**Figure 20:**
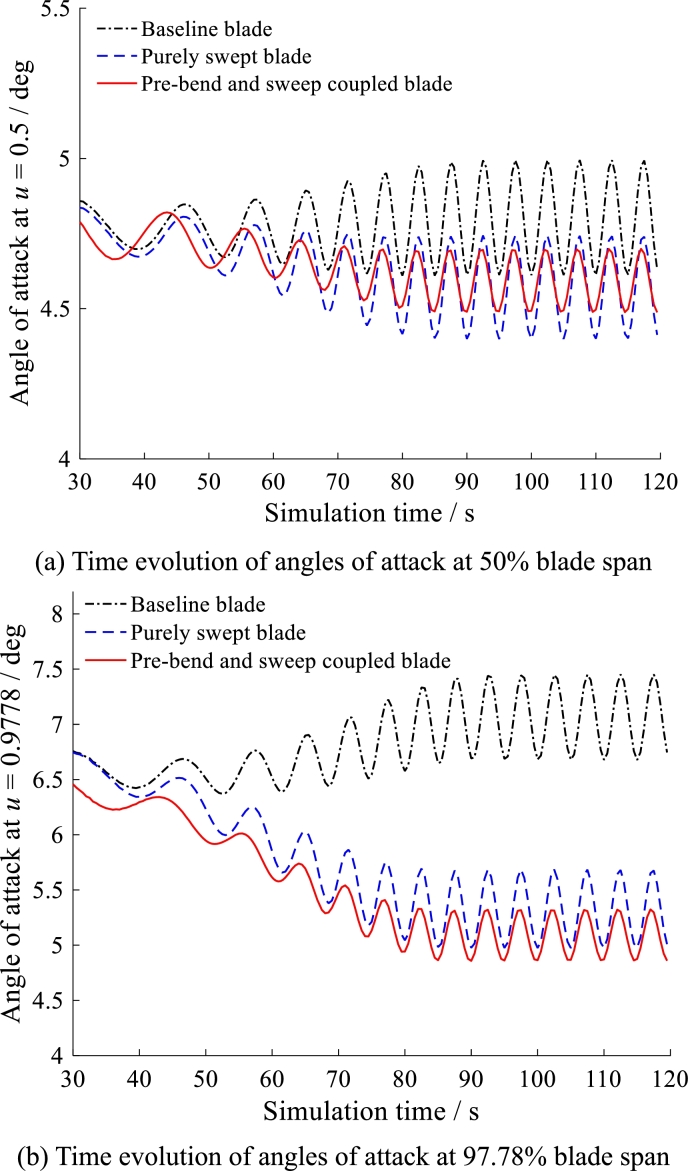
Time evolution of angles of attack at 50% and 97.78% blade span.

**Figure 21:**
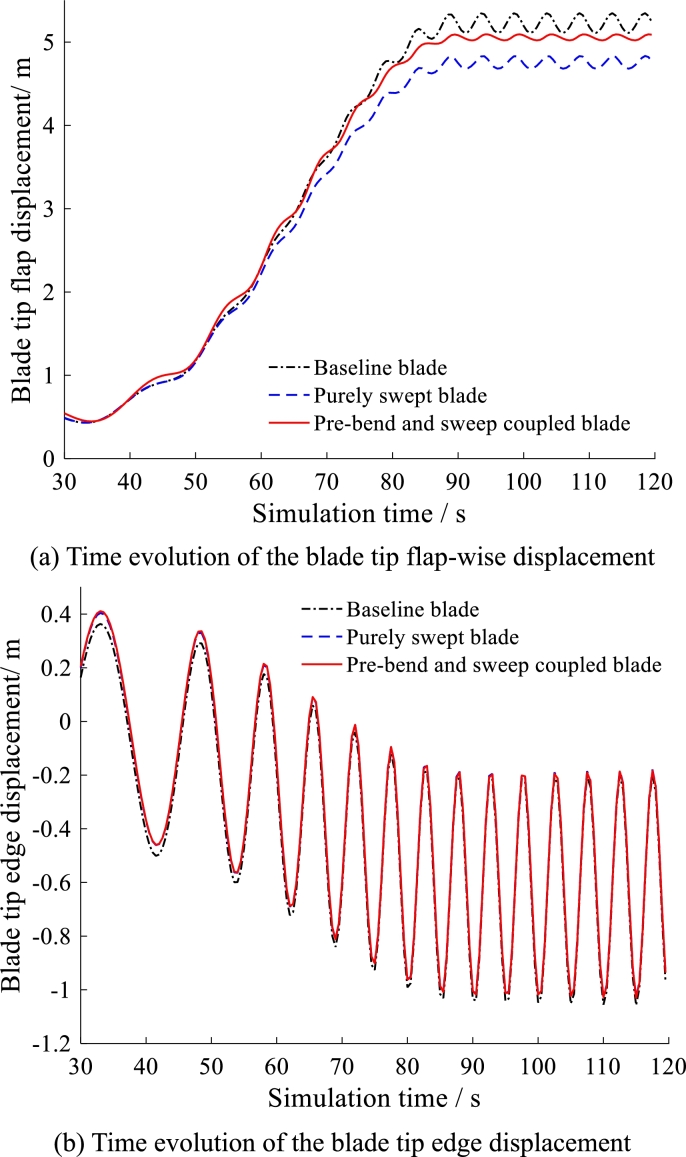
Time evolution of the blade tip displacement.

**Figure 22:**
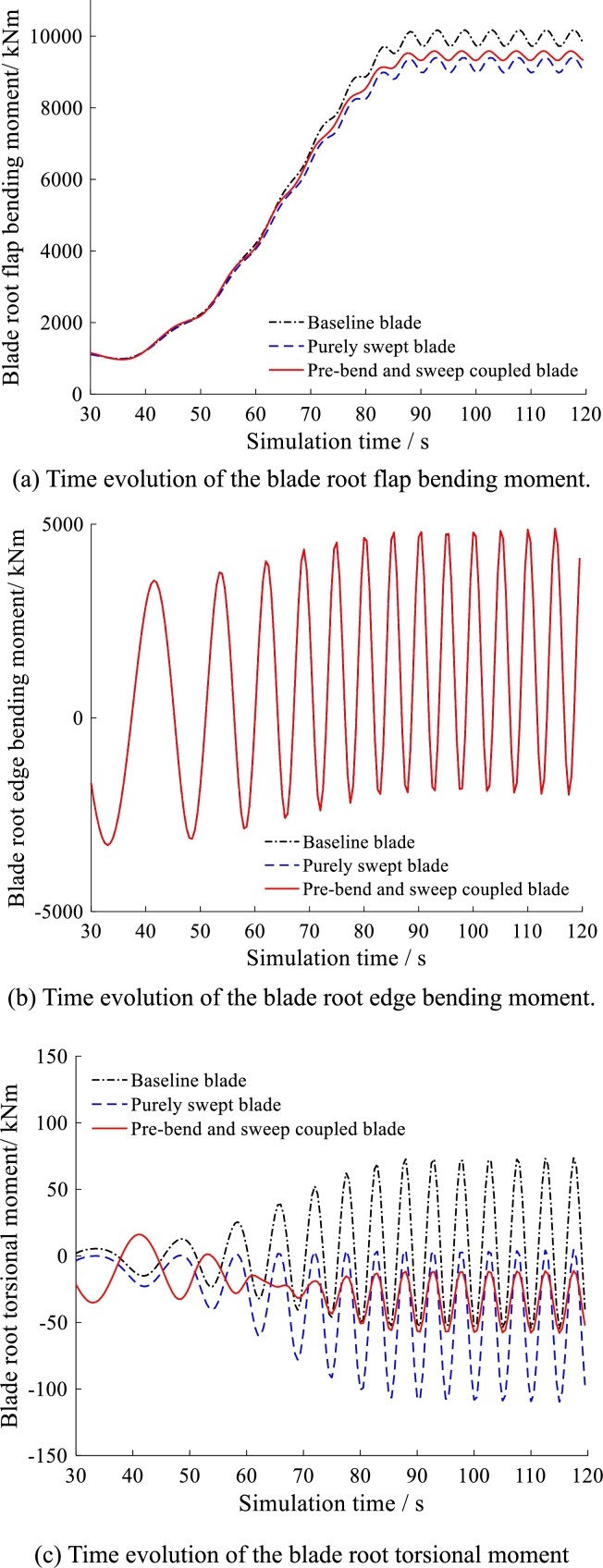
Time evolution of the blade root moment.

The three kinds of blades are simulated under the same operational condition. The simulation results are summarized in Figure 20, Figure 21, Figure 22. All of the results from Fig. 20 to Fig. 22 behave in waveform. This is caused by the circulation of gravity load which is the main source of dynamic fatigue damage during blade rotation.

Fig. 20 show the time evolution of angle of attack at 50% blade span (see Fig. 20 (a)) and at 97.78% blade span (see Fig. 20 (b)). For the variation of angle of attack, both pre-bend/sweep blade and purely swept blade have smaller mean-value than the baseline blade. Moreover, the pre-bend/sweep blade has smaller fluctuation than purely swept blade. It is benefit to alleviate the blade vibration.

The time evolution of the blade tip flapwise displacements is plotted in Fig. 21 (a). The results indicate that the pre-bend/swept blade has larger mean-value but smaller fluctuation than purely swept blade. The larger mean-value does not matter because the tower clearance is improved by its pre-bend. Importantly, the smaller fluctuation has better performance on alleviation of vibration and fatigue load.

For the blade tip edgewise displacement and the blade root edgewise bending moment as shown in Fig. 21(b) and Fig. 22(b), the three kinds of blades have the similar mean-value and fluctuation. This indicates that the pre-bend and swept configuration make slight difference in the blade edgewise displacement and the blade root edgewise bending moment.

Fig. 22 (a) shows the time evolution of blade root flapwise bending moment. It is indicated that both pre-bend/sweep blade and purely swept blade have smaller mean-value when compare to the baseline blade. Besides, the pre-bend/swept blade has larger mean-value but smaller fluctuation than purely swept blade. For the blade root torsional moment as shown in Fig. 22 (c), the purely swept blade has larger absolute-value than baseline blade. It is evidenced that the swept configuration actually induces additional torsional moment, just as Pavese C. and Kim T. et al. [15] studied. However, the newly pre-bend/swept blade has smaller mean-value and lower fluctuation than the purely swept blade. Therefore, it is indicated that the novel pre-bend/swept blade can reduce the blade root torsional moment.

**Figure 23:**
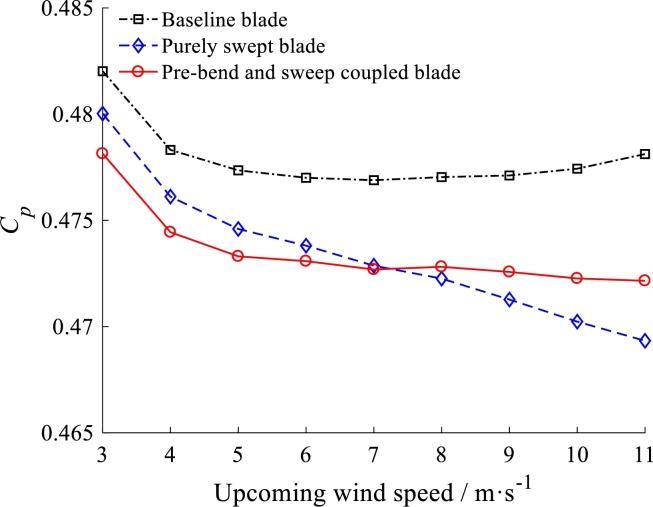
Power coefficients at different wind speed.

The power coefficients which the blade tip speed ratio is about 7 at different wind speed are plotted in Fig. 23. It is investigated that the power coefficients for both pre-bend/swept blade and purely swept blade are slightly lower compare to the baseline blade. In detail, the baseline blade's average of power coefficient is 0.47791. The average of power coefficient for pre-bend/swept blade is 0.47346 which is only reduced by 0.931% than the baseline blade. In addition, the average of power coefficient for the purely swept blade is 0.47339 which is almost same as the pre-bend/swept blade. It is shown that the swept configuration has hardly effect on rotor performance.

In a word, it is concluded that the novel pre-bend/swept blade has more positive attributions., when compared to the purely swept blade and baseline blade, on alleviation of vibration and on reduction of blade root torsional moment.

## 5 Conclusions

An aeroelastic analyzed method of the novel pre-bend/swept blade is presented based on parameterized mathematical model and aeroelastic model. The parameterized model is able to build pre-bend/sweep blade with fully curvilinear axis. The aeroelastic model can solve the problem of the spatial curved and twist blade. Both the structural and the aerodynamic modules can reflect the effects of the blade deformation so that the behavior of the blade bending-torsion coupling can be captured. In order to investigate the geometrical adaptiveness of the pre-bend/swept blade, three kinds of wind turbine blades are designed using the parameterized mathematical method. They are named baseline blade, purely swept blade and pre-bend/swept blade, respectively. A set of aeroelastic simulated analysis is carried out to compare the displacement and loads of the three kinds of blades. It is concluded that the novel pre-bend/swept blade has smaller fluctuation including blade tip displacement and blade root moment, compared to other two kinds of blades. More importantly, compared with the purely swept blade and the baseline blade, the pre-bend/swept blade exhibits smaller mean-value and lower fluctuation of blade root torsion moment. These findings in this paper can effectively alleviate blade vibration and reduce blade fatigue load for the novel pre-bend/swept blade. Additionally, as the geometrical configuration of wind turbine blades is expressed by the control points (see Eq. (1)), it can provide a feasible approach to optimize the pre-bend/swept blade.

In the future research, we will establish an appropriate model of the pre-bend/swept blade to study the aero-elastic characteristic of offshore wind turbine.

## Declarations

### Author contribution statement

Quan Wang: Conceived and designed the experiments; Analyzed and interpreted the data; Wrote the paper.

Cong Hu, Daode Zhang: Analyzed and interpreted the data; Contributed reagents, materials, analysis tools or data; Wrote the paper.

Gang Chen: Conceived and designed the experiments; Wrote the paper.

Fengyun Wang: Performed the experiments; Analyzed and interpreted the data.

### Funding statement

This work was supported by 10.13039/501100001809National Natural Science Foundation of China [51975190] and the Green Industry Science and Technology Leading Plan of 10.13039/501100002948Hubei University of Technology for Excellent Young Scholars [No. XJ2021001201].

### Data availability statement

Data included in article/supp. material/referenced in article.

### Declaration of interests statement

The authors declare no conflict of interest.

### Additional information

No additional information is available for this paper.
